# Amnesia in a Patient with Rheumatoid Arthritis: A Case of Granulomatosis with Polyangiitis

**DOI:** 10.1155/2020/8844951

**Published:** 2020-10-29

**Authors:** Pablo Weilg, Giancarlo Diaz-Zamora, Larry Young

**Affiliations:** ^1^MercyOne North Iowa Medical Center, 1000 4th St SW, Mason City, IA 50401, USA; ^2^University of Miami, Division of Rheumatology, 1120 NW 14th St, Miami, FL 33136, USA

## Abstract

A 56-year-old male with a history of seropositive rheumatoid arthritis complained of polyarthritis and forgetfulness. The initial CT scan of the head without contrast was negative for acute pathologies. However, as he continued to decline despite being on oral prednisone, an MRI of the head was ordered and revealed a subtle small region of acute infarction in the left mammillary body. He was diagnosed with granulomatosis with polyangiitis in light of his positive C-ANCA antibodies, PR3 antibody, and a kidney biopsy, which showed focal necrotizing pauci-immune crescentic glomerulonephritis. Despite undergoing steroid pulse therapy, the patient developed diffuse alveolar damage which finally responded to rituximab infusion.

## 1. Introduction

Granulomatosis with polyangiitis (GPA) is a systemic antineutrophil cytoplasmic autoantibody- (ANCA-) associated vasculitis that is characteristically associated with renal and respiratory disease [1, 2]. Although patients with GPA may develop a wide variety of neurological manifestations, most of them involve the peripheral nervous system and are present in approximately 15% of patients [3, 4]. Central nervous system involvement occurs in less than 10% of patients, with the meningeal disease being the most commonly associated with granulomatous inflammation [5]. Cerebral lesions are very rare and mostly aggressive [6, 7].

## 2. Case Presentation

A 56-year-old male with a past medical history significant for seropositive rheumatoid arthritis (RA) for the past 12 years was seen due to a one-week history of polyarthritis and forgetfulness in the last day. In the emergency department (ED), the physical examination was significant for moderate back tenderness with decreased range of motion, swelling, and tenderness of the metacarpophalangeal joints, wrists, ankles, and left knee. The patient's wife reported he was having trouble recalling recent events. The patient was oriented only to person and place; otherwise, he was alert, following commands, he had a steady gait, and had no focal deficit or cranial nerve involvement. The cardiac examination was normal. Given his recent lumbar laminectomy 2 months prior, a lumbar MRI was ordered in the ED which did not show any acute changes. Furthermore, a CT scan of the head without contrast was negative for stroke, intracranial hemorrhage, or other lesions. Laboratory workup showed a leukocyte count of 12.09 mg/dL and hemoglobin of 11.9 mg/dL. Otherwise, no electrolyte abnormalities were noted, and serum creatinine was 0.89 mg/dL with an estimated GFR >60. No acute phase reactants were tested. The patient was admitted due to a suspected rheumatoid arthritis flare, his prednisone was increased to 15 mg daily, and opioids were given for pain control. Previously, his RA was controlled with chronic use of prednisone 5 mg daily, methotrexate 25 mg SQ, hydroxychloroquine 400 mg daily, and tocilizumab SQ 162 mg for the last 4 years.

**Figure 1:**
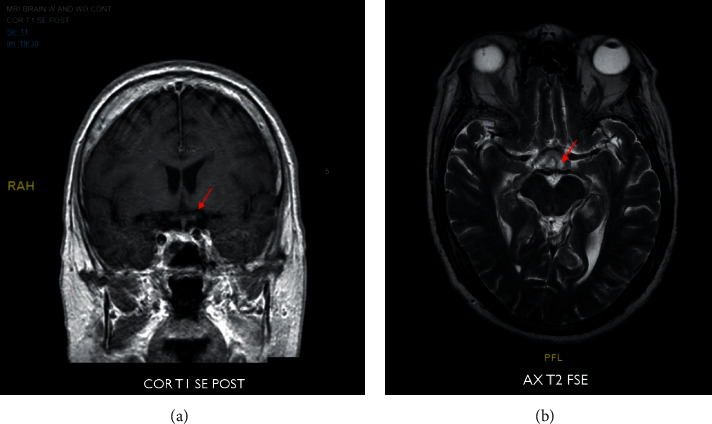
MRI of the brain with and without contrast—small region of focal water restriction at the left mammillary body consistent with a subtle infarct in this area.

On the third day of hospitalization, the patient's polyarthritis showed no improvement, and thus rheumatology was consulted. A nonblanching palpable petechial rash was noted over the patient's lower extremities, and his memory declined as he was struggling to recall same-day events. The patient became oriented only to person, cranial nerves were intact, and no focal deficits were noted, and he was able to follow commands and had no coordination problems. Additional laboratory workup revealed proteinuria and microscopic hematuria with normal creatinine and GFR. An MRI of the brain with and without contrast showed a small region of acute infarction in the left mammillary body (Figure 1).

The patient was started on methylprednisolone IV due to concerns of systemic vasculitis with central nervous system (CNS) and kidney involvement. A CT angiography (CTA) of the head and neck showed bilateral minimal scattered plaques involving the carotid arteries without evidence of high-grade stenosis. A more thorough workup showed normal complement levels, elevated C-reactive protein 15.3 mg/dL, sedimentation rate >130 mm/hr, positive 1 : 512 C-ANCA, positive proteinase 3 antibody >8.0, negative P-ANCA, positive rheumatoid factor 252 IU/mL, negative cyclic citrullinated peptide antibody, negative cryoglobulin, negative dsDNA antibody, and negative ANA. Serology for acute HIV, hepatitis A, B, and C were negative, as well as histoplasma antibodies (Table 1). Echocardiogram showed normal biventricular function with an estimated EF of 55–60% and no signs of pericardial effusion or endocarditis. A kidney biopsy was also planned.

**Figure 2:**
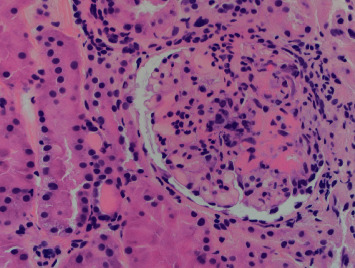
Focal necrotizing crescentic glomerulonephritis on kidney biopsy.

**Figure 3:**
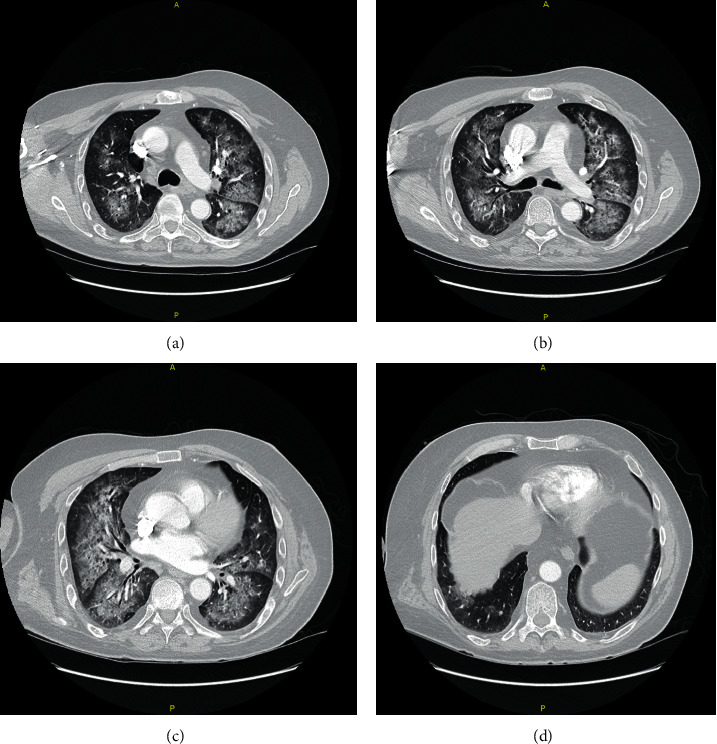
Extensive patchy airspace disease on CT scan of the chest with contrast.

The patient was diagnosed with a C-ANCA-associated vasculitis with CNS involvement, and a treatment plan was established for methylprednisolone 500 mg IV twice a day for 5 days followed by cyclophosphamide infusion once the patient was cleared by infectious disease. The patient's confusion, polyarthritis, and a petechial rash slowly improved after the initial corticosteroid pulse. His kidney biopsy reported focal necrotizing pauci-immune crescentic glomerulonephritis confirming the diagnosis of granulomatosis with polyangiitis (Figure 2). No creatinine elevation or abnormal GFR were seen throughout the patient's hospitalization despite the evident proteinuria and hematuria. After 5 days of methylprednisolone, the patient was discharged on prednisone 60 mg PO daily with plans to start cyclophosphamide within the next few days. Two days later, during his outpatient follow-up with rheumatology, the patient was complaining of hemoptysis and was noted to be in moderate respiratory distress which prompted rapid readmission. A CT scan of the chest showed extensive patchy airspace disease throughout the lungs concerning for diffuse alveolar hemorrhage (Figure 3) which was confirmed with a bedside bronchoscopy. The patient was restarted on methylprednisolone 500 mg IV twice followed by rituximab 1g IV after which he achieved complete remission. The patient continues to be followed as an outpatient and is currently well controlled on rituximab infusion every 4 months.

## 3. Discussion

Cerebrovascular disease in antineutrophil cytoplasmic autoantibody- (ANCA-) associated vasculitis is exceedingly rare and tends to have a poor prognosis [6–9]. Most case reports describe a rapidly progressive clinical course with high mortality due to a subsequent hemorrhage or hemorrhagic conversion [8, 10, 11]. Additionally, in these few patients, ischemic strokes are usually present in the acute phase of the untreated vasculitis and may even be part of the initial presentation. Furthermore, different affected areas of stroke have been described including the bilateral corona radiata, the left pons, the lateral medullary, and the right medulla oblongata [7, 8, 10, 11].

This case presented with polyarthritis and forgetfulness described by his wife as an impairment to recall recent events. The patient had a history of seropositive RA with a positive RF and a negative anti-CCP. He did not improve with prednisone 15 mg daily for the suspected RA flare, and he later presented with a purpuric rash and further decline in his mentation, which prompted the brain MRI that finally showed the small region of acute infarction in the left mammillary body. Interestingly, mammillary bodies play a prominent role in human memory formation and retrieval [12, 13]. Although isolated infarcts to the mammillary bodies are rare, they can result in acute amnesic syndromes including transient global amnesia which is described as a temporary inability to recall recent events [12–14].

The most common etiology of ischemic strokes in patients with granulomatosis with polyangiitis (GPA) is the inflammation of medium-sized intracranial arteries with associated thrombosis, but other mechanisms have been described including cardioembolic events, arterial dissection, and direct invasion from the nasal cavity, which could explain the wide variety of CNS territories affected [11]. Moreover, a study in 2017 has described accelerated atherosclerosis in patients with ANCA-associated vasculitis reporting the presence of carotid atherosclerotic plaques in 30.4% of patients [15].

Ischemic strokes have been reported in GPA patients with no other obvious cardiovascular risk factors [8]. Our patient's cardiovascular risk factors included his history of rheumatoid arthritis and the chronic use of prednisone. On the other hand, he was on methotrexate, which has shown to reduce the overall cardiovascular disease risk in patients with RA, likely by decreasing systemic inflammation, and tocilizumab which could potentially have similar protective effects [16, 17]. Furthermore, we performed a CTA of head and neck which showed bilateral minimal scattered plaques involving the carotid arteries without evidence of high-grade stenosis. Thus, in this particular case, acute vasculitis with associated thrombosis may have played a major role in the development of the stroke rather than any significant atherosclerosis. Also, the lack of significant carotid plaques and a normal echocardiogram makes a cardioembolic event less likely.

Aside from the challenging presentation, induction of complete remission is the goal and expectation of treatment with immunosuppressive therapy in patients with GPA [1, 18]. Our patient clinically responded to the initial 5 days pulse of methylprednisolone, which was tapered to prednisone 60 mg PO daily followed by cyclophosphamide infusion. However, 2 days later, he presented with diffuse alveolar hemorrhage after which rituximab was started immediately, as it was more accessible at the moment of readmission. This clinical course could highlight the aggressive, relapsing, and unpredictable nature of ANCA-associated vasculitis with cerebrovascular disease.

Finally, the development of granulomatosis with polyangiitis in patients with rheumatoid arthritis (RA) is unexpected but has been previously documented [19, 20]. Our patient had an established diagnosis of seropositive RA for 12 years treated with biologics, after which he developed clinical findings suggestive of systemic vasculitis with serological and histological evidence consistent with GPA.

## Figures and Tables

**Table 1:** Laboratory data.

Component	Result	Units
Complete blood cell count		
WBC count	12.09	mg/dL
Red blood cell count	5.16	×10 6/uL
Hemoglobin	11.9	mg/dL
Hematocrit	42	%
Platelet count	370	×10 3/uL

Comprehensive metabolic panel		
Sodium level	135	mMol/L
Potassium level	4.7	mMol/L
Chloride level	99	mMol/L
Carbon dioxide level	26	mMol/L
Anion gap	13	mMol/L
Glucose level	108	mg/dL
BUN	9	mg/dL
Creatinine	0.89	mg/dL
Estimated CrCl AdjBW	123.12	mL/min
Estimated GFR	>60.0	mL/min/1.73 m^2^
Calcium total	8.1	mg/dL
Bilirubin total	0.6	mg/dL
AST/SGOT	33	IU/L
ALT/SGPT	41	IU/L

Chemistry-misc		
Antinuclear antibody (EIA)	Negative	—
dsDNA antibody	Negative 2.0	IU
C-ANCA	Positive 1 : 512	—
P-ANCA	Negative	—
Myeloperoxidase (MPO) antibody	Negative <0.2	—
Proteinase 3 antibody	Positive >8.0	—
Complement antigen C3	160.8	mg/dL
Complement antigen C4	28.6	mg/dL
Cryoglobulin	Negative	—
Cyclic citrullinated peptide antibody	Negative 1.1	unit/mL
Rheumatoid arthritis Qual	Positive 252	IU/mL
Creatinine random urine	199	mg/dL
Protein random urine	70	mg/dL
Histoplasma antibodies	Negative	
1,3-beta-D-Glucan	Negative 37	pg/mL
Hepatitis A (HAAb) antibody IgM	Nonreactive	
Hepatitis A antibody	Nonreactive	
Hepatitis B core antibody IgM	Nonreactive	
Hepatitis B surface antigen	Nonreactive	
Hepatitis C antibody	Nonreactive	
HIV1 p24 and HIV1/HIV2 antibody	Nonreactive	

## Data Availability

All data were obtained from the patient's electronic medical records. Data have been properly displayed to protect patient's identity.
